# Hypoxia, hibernation and Neuroprotection: An Experimental Study in Mice

**DOI:** 10.14336/AD.2018.0702

**Published:** 2018-08-01

**Authors:** Changhong Ren, Sijie Li, Gary Rajah, Guo Shao, Guowei Lu, Rongrong Han, Qingjian Huang, Haiyan Li, Yuchuan Ding, Kunlin Jin, Xunming Ji

**Affiliations:** ^1^Beijing Key Laboratory of Hypoxia Translational Medicine, Beijing 100053, China; ^2^Center of Stroke, Beijing Institute for Brain Disorder, Beijing 100069, China; ^3^Department of Neurosurgery, Wayne State University School of Medicine, Detroit, MI 48201, USA; ^4^Department of Pharmacology and Neuroscience, University of North Texas Health Science Center, Fort Worth, TX 76107, USA

**Keywords:** hibernation, hypothermia, hypoxia, clinical application

## Abstract

Hibernation is a unique physiological state that evolved to survive periods of food shortages. It is characterized by profound decreases in metabolic rate, body temperature and physiological functions. Studies have shown that animals in hibernation can resist neurological damage. Here, we aimed to study whether hypoxia can induce a hibernation-like state in a traditionally non-hibernating animal and whether it is neuroprotective. All procedures were conducted according to international guidelines on laboratory animal safety. Mice C57BL/6 (19-21g) were placed into a 125 mL jar with fresh air and the jar was sealed with a rubber plug. For each run, the tolerance limit was judged by the animals’ appearance for "air hunger”. The animal was removed from the jar as soon as its first gasping breath appeared and was moved to another fresh-air-containing jar of similar volume. This procedure was performed in four runs. The hypoxia exposure significantly decreased oxygen (O_2_) consumption, carbon dioxide (CO_2_) production, respiratory rate and heart rate. Meanwhile, rectal temperature reached a minimum of 12.7±2.56°C, which is lower than a wide range of ambient temperatures. The mimicked hibernation decreased the infarct size in a focal cerebral ischemia mouse model. Our findings suggest the possibility of inducing suspended animation-like hibernation states for medical applications post injury.

Endothermic mammals have the ability to maintain a constant high body temperature (T_b_) over a wide range of ambient temperatures (T_a_) [1]. However, in the cold winter or food-deficient season, some mammals no longer maintain permanent homeothermy and enter a state of hibernation. Hibernation in these heterothermic endothermic animals is characterized by a controlled reduction of T_b_ via the metabolic rate [2, 3]. In the winter, the core body temperature of bears reduce to ~32°C, and the tropical Malagasy lemur, a primate that hibernates in the dry season of the tropics, drops its core body temperature to around 25°C [4]. Arctic ground squirrels can withstand drops in core body temperature to several degrees below freezing point [3]. The above mammals can enter severe hypothermic states during hibernation in which metabolic activities are extremely low, and yet full viability is restored when the animals arouse from such a state [5]. Numerous studies have shown that the hibernation state has great medical benefits for a variety of conditions including several neuroprotective adaptations (i.e. ischemia/reperfusion, trauma, neurodegenerative diseases) [6-9]. Furthermore, hibernation is a promising area for neuroprotection in traumatic brain injury and stroke as suggested by the National Institutes of Health [7]. The hibernation-like states may also be useful in surgical situations and for improving organ preservation for transplantation [10]. Therefore, if non-hibernators can be induced into a hibernation state with similar benefits on metabolism and post hibernation function as true hibernators, this therapy could prove promising for many ailments.

In the 1960s, Dawe et al first induced a hibernation state in a non-hibernator, through injection of serum from torpid animals [11]. From then on, researchers were exploring several methods to induce a hibernation-like state in non-hibernators. How hibernation occurs in non-hibernating animals remains unknown. Hypoxia exposure has been studied for years including detrimental and beneficial effects [12]. Non-lethal hypoxia is well known to lead a drop in T_b_ in newborns and adults of many species including humans [13, 14]. This hypoxia-induced drop in T_b_ and metabolic depression serves a protective role by reducing O_2_ demand, eliminating costly thermogenesis, improving blood O_2_ affinity, and reducing the costs of ventilation [15, 16]. Tattersall et al reported that hypoxia exposure (120 min, 7% O_2_) caused body temperature to drop from the normoxic value of 37.7 to 31.9°C. This was accompanied by a marked metabolic depression (O_2_ consumption was 46% of the normoxic value). These data suggested that controlled hypoxia exposure is a potential method for inducing a hibernation-like state. However, there is no current evidence that a lack of oxygen is a suitable stimulus for entrance into a hibernation-like physiological state [4].

Our study utilizes the development of a unique animal model of hypoxic exposure to assess if non-hibernators could in fact be made to enter a hibernation-like state following controlled hypoxia. We also explored the potential application of the hibernation-like state.

## MATERIALS AND METHODS

### Animal model

All animal experiments were approved by the Institutional Animal Care and Use Committee of Xuanwu Hospital, Capital Medical University, China, and conducted according to guidelines laid out by the National Institutes of Health. Male mice C57BL/6 (19-21g) were used in this study (Vital River Laboratories, Beijing, China). Animals were maintained on a 12-hour light/dark cycle with unlimited access to food and water.

The procedure for hypoxic exposure was performed as previously described [17]. Briefly, the male mice were placed into a 125 mL jar with fresh air, and the jar was sealed with a rubber plug. The tolerance limit was judged by the animals’ appearance of "gasping breath or air hunger’’ for each run. The animal was removed from the jar as soon as its first gasping breath appeared and was moved to another fresh-air-containing jar of similar volume. This procedure was performed once (H1) and repeated two, three and four times (H2 or H3 and H4), respectively. A blank control group with no exposure to hypoxia (H0) was put into the jar without a lid on. The time between the beginning of airtightness and the appearance of the first gasping was termed "tolerance time’’ for each run. Ambient temperature was maintained at 20±0.5°C and concentration of O_2_ was 21% for room air.

### Core body temperature measurement

The rectal temperature of a mouse was measured with a measurement probe immediately after each run (Harvard Apparatus, Holliston, MA, USA).

### Physiological index detection

Blood pressure was measured immediately after removal from the jar using a standard tail cuff (BP-2010A System, Softron, Beijing, CHINA). Heart rate, respiratory rate, O_2_ consumption and CO_2_ production were measured as previously described [18].

### Distal middle cerebral artery occlusion in the mouse

For distal middle cerebral artery occlusion (dMCAO), C57BL/6 (20-22 g) mice were used in this study as described previously [19]. The mice underwent hypoxia exposure for 4 runs at 30 min per run after dMCAO surgery.

### Infarct size measurement

Infarct size was measured with a 2% solution of 2,3,4-triphenytetrazolium-chloride at 24 h after dMCAO as described previously [20].

### Adhesive tape removal test

Sensorimotor functional recovery after stroke was measured at 3 days before dMCAO and at 3 days after dMCAO with the adhesive tape removal test as previously described [21, 22].

### Statistical analysis

Data was expressed as mean ± standard deviation (mean ± SD) and statistical tests were performed with SPSS for Windows, version 17.0 (SPSS Inc.). One-way ANOVA followed by the Student-Newman-Keuls posthoc test was also used for between-groups comparison. In all cases, p<0.05 was the criterion for significance.

**Figure 1.**
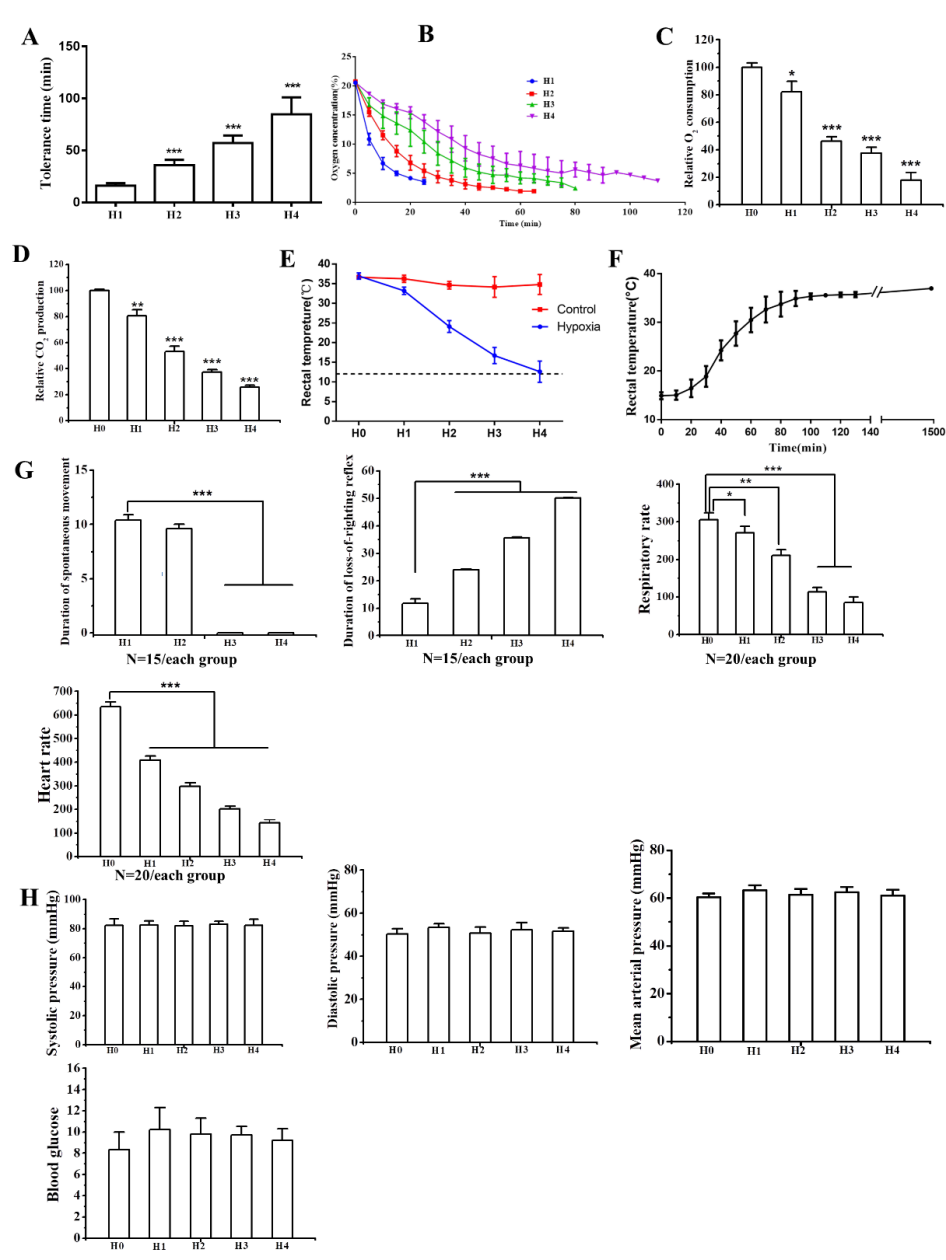
Hypoxia-induced hibernation reduced energy supply and demand. (**A**) Tolerance time in different runs of exposure to hypoxia. ***, *P*<0.001 *vs*. H1, N=13 each group. (**B**) O_2_ concentration in the jar. N=3 each run. (**C**) Relative O_2_ consumption of mice exposed to hypoxia. *, *P*<0.05; ***, *P*<0.001 *vs*. H0, N=12 each group. (**D**) Retative CO_2_ production. **, *P*<0.01; ***, *P*<0.001 *vs*. H0, N=12 each group. (**E**) Rectal temperature at the end of each hypoxic exposure run. *, *P*<0.05; ***, *P*<0.001 *vs*. H0, N=8 each group. (**F**) Recovery of rectal temperature in air after exposure to hypoxia. N=5 each group. (**G**) Hypoxia exposure reduced energy demand. *, *P*<0.05; **, *P*<0.01; ***, *P*<0.001. (**H**) Hypoxia exposure had no effect on blood pressure and blood glucose levels. N=20 each group.

## RESULTS

### Hypoxia exposure reduced energy supply

The tolerance times for when the mice were enclosed in the first jar are shown in Figure 1A; the tolerance time in each jar increased with each run. Oxygen concentration in the sealed jar was gradually reduced to about 3% (Fig. 1B). Oxygen consumption and carbon dioxide production exponentially decreased as exposure run increased. When mice were exposed to hypoxia in the first run, their O_2_ consumption dropped by ~43% and carbon dioxide (CO_2_) output dropped by ~34% (Fig. 1C). By the fourth run, O_2_ consumption dropped by ~80% and CO_2_ output dropped by 68% (Fig. 1D). This drop in metabolic rate was followed by a drop in rectal temperature to ~8°C under T_a_ (Fig. 1E). Rectal temperature was at an average of 36.4°C before the first exposure and decreased to 32.9±1.3°C thereafter (Fig. 1E). Rectal temperature decreased gradually after each run and reached a minimum of 12.7±2.56°C after the fourth run, which was lower than T_a_ (Fig. 1E). After four runs of exposure to hypoxia, the mice were returned to room air, and rectal temperature also returned to normal (Fig. 1F).

**Figure 2.**
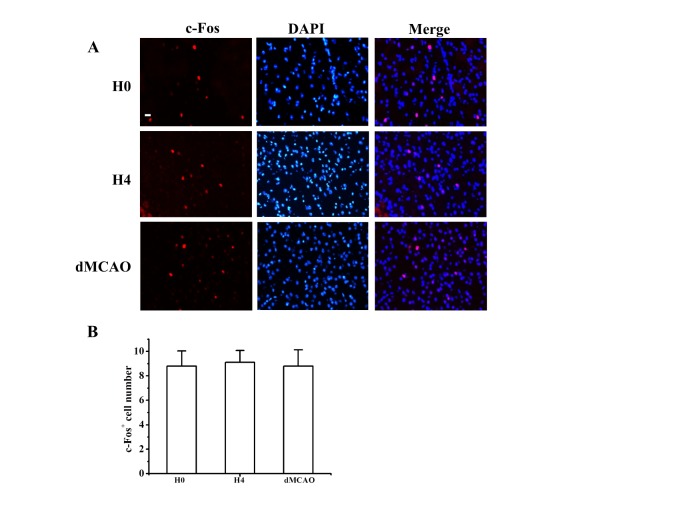
Comparison of c-Fos-positive cell numbers in the hypothalamus before and after hypoxia-induced hibernation (**A**) Representative pictures of c-Fos-positive cells in the hypothalamic region. Scale bar=20 µm. (**B**) Bar graph shows the quantification of c-Fos-positive cells. N=4 each group. N=5 each group.

### Hypoxia-induced hibernation reduced energy demand

One of the characteristics of animals in hibernation is reduced behavioral activity [23]. We first observed the spontaneous movement and righting reflex of the mice in each run. The duration of spontaneous movement of the mice was 10.4 min on average after hypoxia exposure at the first run (Fig. 1G). No recovery in spontaneous movement was seen after the third run. After the fourth run, the animals moved around minimally (Fig. 1G). Next, we analyzed the duration of loss-of-righting reflex (LORR) in each run [24, 25]. The duration of LORR increased as the number of hypoxic exposure runs increased (Fig. 1G). The duration of LORR was 50.1 min on average during the fourth run oxygen exposure (Fig. 1G).

Heart rate and respiratory rate reduced after each run (Fig. 1G). Respiratory rate was 305 breaths per min at the beginning of the first run and decreased by 71% at the end of the fourth run (Fig. 1G). Similarly, heart beat was reduced by 75% (Fig. 1G).

### Hypoxia-induced hibernation had no effect on blood pressure and blood glucose

Blood pressure remained at the same level without decreasing throughout the four runs (Fig. 1H). Blood glucose level was increased in H1 but remained at the same level without continuing to increase in H2, H3 and H4 (Fig. 1H).

**Figure 3.**
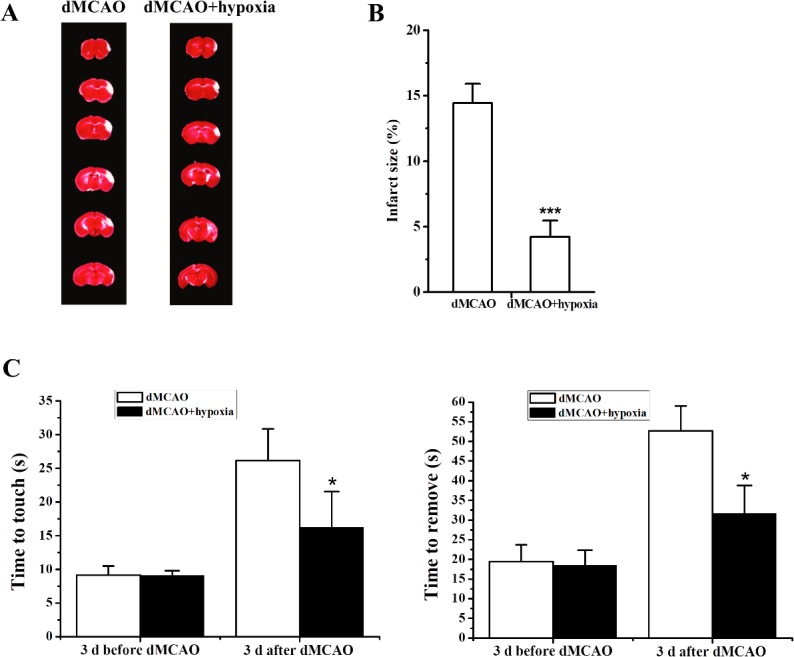
Hypoxia-induced hibernation reduced infarction (**A**) Representative infarcts stained using 2,3,4-triphenytetrazolium chloride (TTC) from the dMCAO only group and dMCAO mice treated with hypoxia. (**B**) Average infarct sizes. ***, *P*<0.001, *vs*. dMCAO only. N=7 each group. (**C**) Hypoxia significantly improved sensorimotor functions after dMCAO, *, *P*<0.05 *vs*. dMCAO only. N=12 each group.

### The effect of hypoxia-induced hibernation on neuronal activity

It is well known that c-Fos serves as an index for neuronal activity [26]. Therefore, we investigated the number of c-Fos positive cells between the H0 and H4 groups in the hypothalamus as this is the thermoregulatory center in the brain with multiple nerve nuclei [27]. Our results showed that there was no significant difference between the two groups (Fig. 2), suggesting that the decline in body temperature was not a failure of body temperature regulation, but possibly an active expansion of the adaptive capacity.

### Hypoxia-induced hibernation reduced cerebral ischemia induced injury

To explore whether the hypoxia-induced hibernation state had the potential for neuroprotection after large vessel occlusion, we performed focal cerebral ischemia on mice using the dMCAO method. Mice were exposed in the sealed jar for four runs at 30 minutes after dMCAO surgery. The dMCAO mice that underwent hypoxia exposure (N=7) averaged 4.2±1.22% infarct volumes compared with 14.4±1.49% infarct volumes in the dMCAO only (N=7) group (Figs. 3A and B). This 71% reduction proved to be significant (*P*<0.001). Neurological function was evaluated using the adhesive tape removal test. Hypoxia exposure treatment significantly improved neurological performance in the adhesive tape removal test after dMCAO as manifested by a consistent reduction in the number of times to contact and then remove the tape from the contralateral limb (Fig 3C). One study reported that MCAO may affect hypothalamic function [28]. We therefore compared the number of c-Fos positive cells among H1, H4 and dMCAO mice. Our results showed that there were no significant differences among them (Fig. 2), suggesting that dMCAO did not affect hypothalamic function and that hypoxia-induced hibernation state had a direct effect on neuroprotection.

## DISCUSSION

In this study, we developed a method that enabled mice to enter a hibernation-like state with the rectal temperature dropping to ~8°C under T_a._ The mimicked hibernation had a neuroprotective role in preventing cerebral ischemia induced injury due to occlusion of the distal MCA.

In 1960s, Dawe *et al* first induced hibernation state in a non-hibernator, through the injection of serum from torpid animals [11]. To date, there are several molecules that can result in reversible severe hypothermia when administered to small mammals [5]. Roth et al utilized hydrogen sulfide gas in 2005 to induce hypothermia in lab animals. The average core body temperature of these mice reached a minimum of 15°C in an ambient temperature of 13°C [29]. After receiving the glycolytic inhibitor 2-deoxyglucose, hamsters readily underwent a decrease in T_b_ below 30°C despite being kept in long photoperiods [30]. Mice that received 5′adenosinemonophosphate (5′-AMP) were severely hypothermic, with T_b_ as low as 25°C when kept in T_a_ of about 23-24°C [31]. Clinically, phenothiazine drugs, chlorpromazine and promethazine can induce mild hypothermia of 32-34°C [7].

Hypoxia is well known to reduce the T_b_ of mammals although the mechanism remains unclear. Tattersall et al reported that after exposure for 2 hours at 7% oxygen concentration, T_b_ only reduced to 31.4°C [32]. The fall in temperature that occurs during hypoxia is the result of a reduction in the activation of thermogenic mechanisms [32]. However, the previous study has demonstrated that hypoxia can reduce body temperature to 31°C but could not reach the body temperature while hibernating [33]. Our study showed that repetitive hypoxia exposure can drive the mouse into severe hypothermia 12.7±2.6°C, which is lower than an ambient temperature of 20°C. However, our method of inducing low temperatures is different from this method of placing animals in hypobaric chambers of fixed and known concentrations. The process of our method is repetitive hypoxic exposure, and in each hypoxic exposure run, the O_2_ concentration in the jar is gradually reduced to about 3%. In this study, the exact mechanism of hypothermia is not known. Further study into the physiological and molecular regulators driving the controlled hypoxia-induced hibernation is therefore necessary.

We then explored whether the hypoxia-induced hibernation state is neuroprotective. Our results showed that hypoxia exposure significantly reduced focal cerebral ischemia induced injury in a dMCAO model. Whether this protection via the hibernation method can be applied to other fields remains to be explored. If humans can be induced into a hibernation state, there will be significant clinical value. Hibernation therapy can have broad ranging protective effects from post ischemic infarct tissue preservation to giving transplant patients more time to await matches.

It is interesting to note that whether the hypoxia is preconditioning (before the detrimental event) or after as in our model, controlled hypoxia results in protective effects [18]. Preconditioned hypoxic animals’ cerebrospinal fluids (CSF) have previously been shown to be neuroprotective for cortical rat neurons in culture, suggesting that endogenous factors exist in the CSF. The mechanism of protection between pre-hypoxic conditioning and post-hibernation states are likely similar. Altitude training and hypoxic conditioning amongst endurance athletes are other avenues where controlled hypoxia is utilized today. Elite swimmers training at a higher altitude have been shown to have increased hemoglobin mass, increased lactate thresholds, and faster time, suggesting a more efficient homeostasis. Erythropoietin is elevated in altitude training as well and has been found to be neuroprotective by others [34].

Post cardiac arrest patients are traditionally cooled, following the results of the hypothermia after cardiac arrest study in which both mortality and favorable neurological outcome were found to be better with mild hypothermia 32-34°C for 24 hours following cardiac arrest. Antidotal cases of near drowning in frigid water with neurological recovery despite prolonged circulatory arrest also support hypothermia-induced neuroprotection [35]. Furthermore, circulatory arrest and hypothermia are routinely used for surgical procedures [36]. Mild hypothermia has shown some promise in humans after acute ischemic stroke via cooling blankets/cold saline infusion [37] and endovascular selective catheter infusion of cold saline was determined to be safe [38].

While it might seem counterintuitive to place an animal undergoing a large vessel occlusion into an increasingly hypoxic environment, it is the slow controlled aspect of the hypoxia that likely results in clinical benefit. In fact, the opposite has been trialed in humans, with hyperbaric oxygen supplied to stroke patients and the authors concluded there was no benefit and could even possibly harm patients [39]. However, hyperbaric oxygen therapy in mice has been found to be neuroprotective when used as a pre-conditioning therapy [40]. Thus, many questions remain unanswered. Free divers and mountaineers are two populations of people exposed to acute and chronic hypoxia, respectively. Arterial O_2_ partial pressures as low as 19-23 mmHg and CO_2_ levels of 16-61 mmHg are tolerated via large increases in CBF [41]. However, this increase in CBF is likely necessary for the maintenance of consciousness and motor function, in a controlled hibernation state, such an increase in CBF would probably not be necessary. Human hibernation has recently been described as an epiphenomenon to HIV-related injury to the hypothalamus and thalamus in which the patient experiences seasonal hypothermia (nadir 31°C), bradycardia, encephalopathy, and hypersomnolence made better with external warming [42]. Mild therapeutic hypothermia (32°C-35°C) has been recognized as an effective neuroprotectant in experimental stroke models [43-45]. Our present study showed that severe hypothermia induced by hypoxia still has the neuroprotective role in the stroke model. Although the mechanism need to be further explored, the concept-proofed neurorpteciton would be used in a wider range of fields.

Taken together, our findings deserve a closer look with more animals to determine the best regimen for inducing hibernation. We believe that with the continuous advancement of science and technology, it will not be before long where humans can enter hibernation simply, safely, and reliably.
